# AMF promotes the structure and metabolic activity of rhizosphere soil microbial communities in areca/vanilla intercropping system under nitrogen-limited conditions

**DOI:** 10.3389/fmicb.2025.1657672

**Published:** 2025-09-23

**Authors:** Huifa Zhuang, Zhuo Feng, Yunbin Hu, Ziwei Ning, Qingyun Zhao, Hui Wang, Yizhang Xing, Ang Zhang

**Affiliations:** ^1^Spice and Beverage Research Institute, Chinese Academy of Tropical Agricultural Sciences, Wanning, Hainan, China; ^2^Ministry of Agriculture and Rural Affairs, Key Laboratory of Genetic Resource Utilization of Spice and Beverage Crops, Wanning, Hainan, China; ^3^Hainan Key Laboratory of Genetic Improvement and Quality Regulation for Tropical Spice and Beverage Crops, Wanning, Hainan, China; ^4^College of Tropical Crops, Yunnan Agricultural University, Pu’er, Yunnan, China

**Keywords:** areca, vanilla, arbuscular mycorrhizal fungi, nitrogen reduction, rhizosphere microenvironment

## Abstract

This study systematically investigated the response mechanisms of the rhizosphere microenvironment and physiological metabolism in an areca/vanilla intercropping system under three nitrogen reduction levels (conventional, 30% reduction, and 60% reduction) combined with inoculation of two arbuscular mycorrhizal fungi (AMF). The results showed that under 30% nitrogen reduction, inoculation with *Claroideoglomus etunicatum* significantly increased soil hydrolytic nitrogen by 26.29% (*P* < 0.05), this change directly drives the increase in nitrate reductase activity and photosynthetic pigment synthesis in plant leaves by optimizing the microenvironment of root nitrogen supply. *Funneliformis mosseae* increased soil organic matter by 10.93% (*P* < 0.05) by enriching the rhizosphere carbon source pool, reshaping the microbial interaction network, and indirectly promoting physiological metabolism related to root nutrient absorption. AMF exhibited species-specific regulation of soil enzyme activities. For instance, under conventional fertilization, *Claroideoglomus etunicatum* increased phosphatase activity by 25.68% (*P* < 0.05) by enhancing the biochemical microenvironment of organic phosphorus mineralization in the rhizosphere, thereby providing substrates for plant phosphorus metabolism. While under 60% nitrogen reduction, *Funneliformis mosseae* boosted urease activity by 64.46% (*P* < 0.05). This response stems from its induction of the enrichment of urease-producing bacterial communities in the rhizosphere, accelerating the conversion of organic nitrogen to alleviate nitrogen metabolic stress in plants under low nitrogen stress. Additionally, nitrogen reduction combined with AMF inoculation significantly promoted the accumulation of glomalin-related soil protein (GRSP). At 60% nitrogen reduction treatment, compared with the control group without AMF inoculation, the treatment group inoculated with *Funneliformis mosseae* showed a 5.16% (*P* < 0.05) increase in GRSP. This study demonstrates that AMF optimizes rhizosphere nutrient cycling (e.g., AMF (C.e) significantly increases hydrolytic nitrogen by 26.29% (*P* < 0.05) under 30% nitrogen reduction) and improves soil structure (e.g., AMF (F.m) promotes GRSP accumulation by 5.16% (*P* < 0.05), providing a theoretical basis for reducing fertilizer application and promoting sustainable intensification of tropical intercropping systems.

## 1 Introduction

Areca (*Areca catechu* L.) as a perennial evergreen tree of the palm family, has typical tropical plant characteristics, and its growth conditions are high temperature and high humidity environment, the species is not only an important landscape plant in the tropics, but also listed as the first of the four major southern medicines because of its outstanding medicinal value, and organs such as fruits, seeds, and flowers have medicinal efficacy (Wang et al., 2025). Currently in Hainan, China, areca cultivation has formed a large-scale industry, which has become an important pillar of local farmers’ economic income. With the sustained high market price of betel nut in recent years, the application of nitrogen fertilizer has shown a significant increasing trend, but empirical studies have shown that when the nitrogen application exceeds the threshold, the yield does not show a linear increase, but tends to stabilize. It is of concern that the current over-application of nitrogen in betel nut plantations leads to a utilization rate of less than 30%, and nitrate leaching due to over-application has posed a serious pollution threat to the groundwater system (Bhat et al., 2007).

Vanilla (*Vanilla planifolia* Andrews), as a perennial tropical vine of the genus Vanilla in the family of Orchidaceae, is known as the “King of Natural Food Flavorings” for its unique aromatic value (Gu et al., 2024). Its pods can extract more than 250 kinds of volatile aromatic substances after professional aroma treatment, which are widely used in high-end food, cosmetics and pharmaceutical fields (Rodríguez-López and Martínez-Castillo, 2019). Modern pharmacological research has confirmed that vanilla orchids have the health effects of tonifying the kidneys and strengthening the stomach, eliminating flatulence and detoxification, etc., and have been included in the Pharmacopeias of many countries in Europe and the United States (Anuradha et al., 2013). This crop has the biological characteristics of shallow aerial root system, and its shade-loving growth habit is complementary to the semi-shade environment of betel nut plantation. The intercropping model study showed that the betel nut/vanilla orchid composite system could not only realize the efficient use of land resources (significant increase in the land equivalent ratio), but also increase the nitrogen uptake efficiency by 21.4% and nitrogen utilization efficiency by 18.9% through the effect of root interactions, and the systematic nitrogen fertilizer utilization reached an optimized level of 45.3% (Sujatha and Bhat, 2010). This ecologically intensive cultivation model provides a new practical path for the sustainable development of agriculture in the tropics.

Arbuscular mycorrhizal fungi (AMF) is an important part of the soil microbial community and can establish a good symbiotic relationship with most plants (Duan et al., 2024). By applying fertilizer to make AMF symbiosis to promote shallow-rooted plants to obtain more nutrients, it is important to improve the current crop production of excessive fertilization, not only can effectively improve the soil crust, but also can improve the ability of soil water and fertilizer, reduce the soil pathogenic fungi, and promote the growth of beneficial fungi (Sun and Shahrajabian, 2023). It was found that intercropping pattern significantly increased the biomass and nitrogen uptake of betel nut and vanilla orchid under the same nitrogen application treatment, but there was biological nitrogen fixation, while the soil total nitrogen content was not reduced. This phenomenon may be attributed to the neglect of the role of mycorrhizal fungi in the nitrogen cycle of adjacent soil ecosystems in previous studies. For instance, traditional research holds that the functions of AMF mainly focus on absorbing minerals such as phosphorus and potassium, thereby improving plant nutritional status (Jiang et al., 2021). However, on the one hand, AMF can enhance the overall vitality of plants, promoting the secretion of some plant photosynthates into the rhizosphere through AMF, thereby supporting the nodulation and nitrogen-fixation activities of nitrogen-fixing bacteria and exerting a positive feedback effect on the nitrogen uptake efficiency of plants (Wahab et al., 2023). On the other hand, AMF improve soil structure, reduce nitrogen leaching and volatilization, and retain more nitrogen in the rhizosphere, which further enhances the nitrogen supply from the soil ecosystem to plants (Xiaomei et al., 2024). Therefore, AMF in agricultural intercropping systems may indirectly regulate the structure of microbial communities, thereby promoting the absorption efficiency of plants for diverse nutrients. However, current research primarily focuses on the effects of intercropping patterns on crop yield and quality, with relatively little attention paid to the mechanisms by which nitrogen fertilizer and AMF synergistically regulate the structure and metabolic activity of the rhizosphere soil microbial community in intercropping systems. In particular, the systematic analysis of the synergistic effects between AMF and fertilizers remains to be further refined. In this study, a pot experiment was conducted to monitor the impacts of inoculation with different AMF strains under reduced nitrogen fertilizer application rates on the rhizosphere microenvironment, soil enzyme activities, and rhizosphere microbial community structure in the intercropping system. The objective was to clarify the regulatory mechanisms through which nitrogen fertilizer and AMF synergistically improve soil structure, activate soil mineral nitrogen nutrients, and promote the efficient absorption and utilization of nitrogen in the intercropping system. The findings of this research will provide a theoretical basis for realizing nitrogen fertilizer reduction in intercropping fields and improving the quality of fruits and vegetables.

## 2 Materials and methods

### 2.1 Materials

The test materials came from the experimental demonstration base of the Institute of Spices and Beverages, Chinese Academy of Tropical Agricultural Sciences, Wanning City, Hainan Province, and the test crop varieties were betel nut No. 1 and vanilla orchid No. 3. Betel nut No. 1 was selected and bred by the Institute of Coconut Research of the Chinese Academy of Tropical Agricultural Sciences and is characterized by early fruiting, high yield and good quality; Vanilla orchid No. 3 was selected and bred by the Institute of Spices and Beverages of the Chinese Academy of Tropical Agricultural Sciences, and is characterized by stable heritability, high yield and good quality. It is characterized by genetic stability, high yield and good quality. The young sets of *Claroideoglomus etunicatum* and *Funneliformis mosseae* were developed by the Institute of Plant Nutrition and Resources, Beijing Academy of Agriculture and Forestry. The soil used in the experiment was selected from the cultivated soil of high-yield fields, naturally dried and sieved, then packed in pots, mixed well with fertilizer before sowing, and put into polyethylene plastic buckets with a diameter of 50 cm and a height of 45 cm, with 40 kg of air-dried soil in each bucket.

### 2.2 Methodologies

#### 2.2.1 Experimental treatments

The test site was located in the experimental base of the Institute of Spices and Beverages of the Chinese Academy of Tropical Agricultural Sciences, and the test soil was a sandy loam, with the nutrient contents of 16.73 g kg^–1^ organic matter, 0.68 g kg^–1^ total nitrogen, 113.16 mg kg^–1^ alkaline nitrogen, 14.21 mg kg^–1^ quick-acting phosphorus, 91.33 mg kg^–1^ quick-acting potassium. A two-factor inoculation × fertilization was set up with three treatments, namely (1) no inoculation control (NAM), (2) inoculation of *Claroideoglomus etunicatum* (AMF(C.e)), and (3) inoculation of *Funneliformis mosseae* (AMF(F.m)). Each treatment was set at three levels according to the amount of fertilizer applied: conventional fertilization (CK), reduced fertilization by 30% (A), and reduced fertilization by 60% (B), where the conventional fertilization was used as a control, with five replicates of five pots per treatment, for a total of 225 pots. For potted vanilla plants under normal fertilization, organic fertilizer was applied at a rate of 50 g⋅kg^–1^ along with CH_4_N_2_O (urea) applied at 20 g⋅kg^–1^. For the 30% reduced fertilization treatment, organic fertilizer was applied at 35 g⋅kg^–1^ and CH_4_N_2_O at 14 g⋅kg^–1^. For the 60% reduced fertilization treatment, organic fertilizer was applied at 20 g⋅kg^–1^ and CH_4_N_2_O at 8 g⋅kg^–1^. Seedlings were inoculated with 5 g of mycorrhizal agent 1 month after planting, and fertilizer treatments were applied after 3 months of routine management. Samples were collected half a year after the fertilization treatment was applied.

The plants were uprooted, and the loose soil around the inter-root was taken and divided into three portions after removing debris; the first portion was used for microbiota analysis; the second portion was air-dried and then passed through 18 and 70 mesh subsampling sieves, put into a sample bag, and stored in a dry place; the third portion was passed through a 10 mesh subsampling sieve, put into sterile EP tubes, and quickly put in liquid nitrogen for preservation and stored in a refrigerator at – 80 °C.

#### 2.2.2 Measurement indicators and methods

(1)    Determination of physical and chemical properties of soil: Soil pH was measured with a pH meter (Metler Toledo) at a soil-water ratio of 1: 2.5; Organic matter was determined by potassium dichromate volumetric method with external heating; Hydrolyzable nitrogen was determined by the alkaline diffusion method; Effective phosphorus was determined by 0.5 mol L^–1^NaHCO_3_ leaching-molybdenum blue colorimetric method; Fast potassium was extracted by NH_4_OAc and determined by flame photometry.
(2)    Determination of soil microbial activity: Phosphatase was determined by the colorimetric method using disodium benzene phosphate; Urease was determined by the sodium phenol-sodium hypochlorite colorimetric method; Sucrase was determined by a colorimetric method using 3,5-dinitrosalicylic acid; Cellulase was determined by a colorimetric method using 3,5-dinitrosalicylic acid; Catalase was determined by titration with potassium permanganate.
(3)    Determination of Glomalin-related soil protein: Glomalin-related soil protein (GRSP) content (μg/g) = C × V × N/W
C:    Concentration derived from the standard curve (μg/mL); V: total volume of extract (mL); N: dilution factor; W: sample weight (g).
(4)    Extraction and sequencing of soil total DNA: Soil microbial DNA was extracted three times from 0.5 g of fresh soil using the EZNA^®^ Soil DNA Extraction Kit (Omega Bio-tek, Inc., USA). The purity and quality of the genomic DNA were determined via 0.8% agarose gel electrophoresis. For bacteria, the barcoded primers 338F (5′-ACTCCTACGGGAGGCAGCAG-3′) and 806R (5′-GGACTACHVGGGTWTCTAAT-3′) were used to amplify the corresponding fragment of the V3-V4 region of the bacterial 16S rRNA gene. The fragment length of the amplification products was verified by 2% agarose gel electrophoresis. After pooling the amplification products, a clone library was constructed based on the results of quantitative detection. The loading amount of each library was calculated according to the library validation results, and paired-end sequencing was performed using the Illumina MiSeq high-throughput sequencing platform (Zhao et al., 2024).
(5)    Bioinformatics analysis: In this study, FLASH 1.2.11 software and the QIIME 1.9.1 quality filtering tool were employed to obtain valid sequences by merging the paired-end sequencing data of the original DNA fragments. Unique sequences with ≥ 97% similarity were clustered into operational taxonomic units (OTUs) using UPARSE 7.0.1090 software. Each OTU was annotated via MOTHUR 1.30.2 using the small subunit (SSU) rRNA SILVA database, and the sample with the lowest data volume was used as the reference for data normalization. Indices of soil microbial community richness and diversity were all calculated using QIIME 1.9.1 software (Zhao et al., 2024).

#### 2.2.3 Data processing and statistical methods

Two-way analysis of variance (ANOVA) was performed on all data to determine the differences in the effects of nitrogen application treatments and AMF inoculation on soil physicochemical properties, soil microbial enzyme activities, and soil microbial abundance. One-way ANOVA was conducted to clarify the effects of different AMF species or different nitrogen application rates on the abovementioned indicators, with Duncan’s test used as a *post hoc* test for the above ANOVAs. All data passed the normality test before being subjected to ANOVA. Principal component analysis (PCA) was applied to identify the effects of different nitrogen application treatments and AMF inoculation on soil microbial diversity (β-diversity). Data processing was performed using SAS V8 and R 4.1.1 software. Graphs were generated using Origin 2021 (OriginLab, Northampton, USA).

## 3 Results and analysis

### 3.1 Effect of AMF on the physicochemical properties of plant inter-root soil under reduced fertilization

As shown in Table 1, in uninoculated (NAM), soil pH and effective phosphorus increased and then decreased with the reduction of fertilizer application, and soil organic matter, hydrolyzable nitrogen, and quick-acting potassium showed a decreasing trend with the reduction of fertilizer application, compared with the conventional fertilization (CK). In inoculated AMF (C.e), soil hydrolyzable nitrogen was elevated and then decreased with decreasing fertilizer application, and soil pH, organic matter, effective phosphorus, and quick-acting potassium showed a decreasing trend with decreasing fertilizer application, as compared to conventional fertilization (CK). In the AMF (F.m) inoculated group, soil pH, organic matter, and effective phosphorus increased with decreasing fertilizer application, soil hydrolyzable nitrogen decreased with decreasing fertilizer application, and soil quick-acting potassium first increased and then decreased with decreasing fertilizer application, as compared to conventional fertilization (CK). Soil pH, organic matter, hydrolyzable nitrogen, effective phosphorus and fast-acting phosphorus were reduced when inoculated with AMF (C.e and F.m) as compared to no inoculation (NAM) at conventional fertilization (CK) level, and soil hydrolyzable nitrogen was increased by 26.29% when inoculated with AMF (C.e) as compared to no inoculation (NAM) at reduced 30% fertilization (A) level. Soil organic matter and quick-acting potassium content increased by 10.93 and 2.38%, respectively, when inoculated with AMF (F.m) as compared to non-inoculated (NAM). At reduced 60% fertilization (B) level, soil quick potash content increased by 6.85% when inoculated with AMF (C.e) as compared to non-inoculated (NAM). Soil pH and organic matter content increased by 1.59 and 23.19% when inoculated with AMF (F.m) as compared to non-inoculated (NAM).

**TABLE 1:** Effect of different fertilization levels of AMF on physicochemical properties of rhizosphere soil.

Treatments	Fertilization level	pH H_2_O:W 1:2.5	Organic matter (g^•^kg^–1^)	Hydrolytic nitrogen (mg^•^kg^–1^)	Available P (mg^•^kg^–1^)	Available K (mg^•^kg^–1^)
NAM	CK	6.6 ± 0.2^ab^	42.83 ± 5.5^a^	229.67 ± 62.32^a^	18.13 ± 6.59^ab^	262.67 ± 66.94^a^
	A	6.7 ± 0.1^a^	37.5 ± 6.06^ab^	174.2 ± 94.09^a^	19.97 ± 5.79^a^	252.33 ± 88.38^a^
	B	6.27 ± 0.58^abc^	33.2 ± 7.77^ab^	220.67 ± 90.8^a^	9.59 ± 7.56^abc^	219 ± 27.62^a^
AMF(C.e)	CK	6.47 ± 0.06^abc^	37.77 ± 5.3^ab^	184.67 ± 42.16^a^	15.03 ± 6.45^abc^	248 ± 58.66^a^
	A	6.13 ± 0.5^abc^	36.93 ± 4.27^ab^	220 ± 21^a^	9.46 ± 5.2^abc^	242.67 ± 41.65^a^
	B	6.07 ± 0.15^bc^	31.6 ± 5.28^b^	199.67 ± 11.5^a^	6.39 ± 4.52^c^	234 ± 16.09^a^
AMF(F.m)	CK	6 ± 0.3^c^	34.5 ± 3.64^ab^	226.67 ± 84.72^a^	8.4 ± 4.52^bc^	228.33 ± 48.06^a^
	A	6.57 ± 0.06^abc^	41.6 ± 2.81^ab^	171 ± 53.02^a^	14.33 ± 6.76^abc^	258.33 ± 97.45^a^
	B	6.37 ± 0.15^abc^	40.9 ± 7.16^ab^	184.33 ± 67.34^a^	5.9 ± 4.71^c^	177.67 ± 79.2^a^

NAM, no inoculation control; AMF(C.e), inoculation of *Claroideoglomus etunicatum*; AMF(F.m), inoculation of *Funneliformis mosseae*; CK, conventional fertilization; A, reduced fertilization by 30%; B, reduced fertilization by 60%. Data are presented as mean ± standard errors. Different lowercase letters indicate significant differences at the 5% level.

### 3.2 Effect of AMF on soil catalase activity under reduced fertilization

**FIGURE 1:**
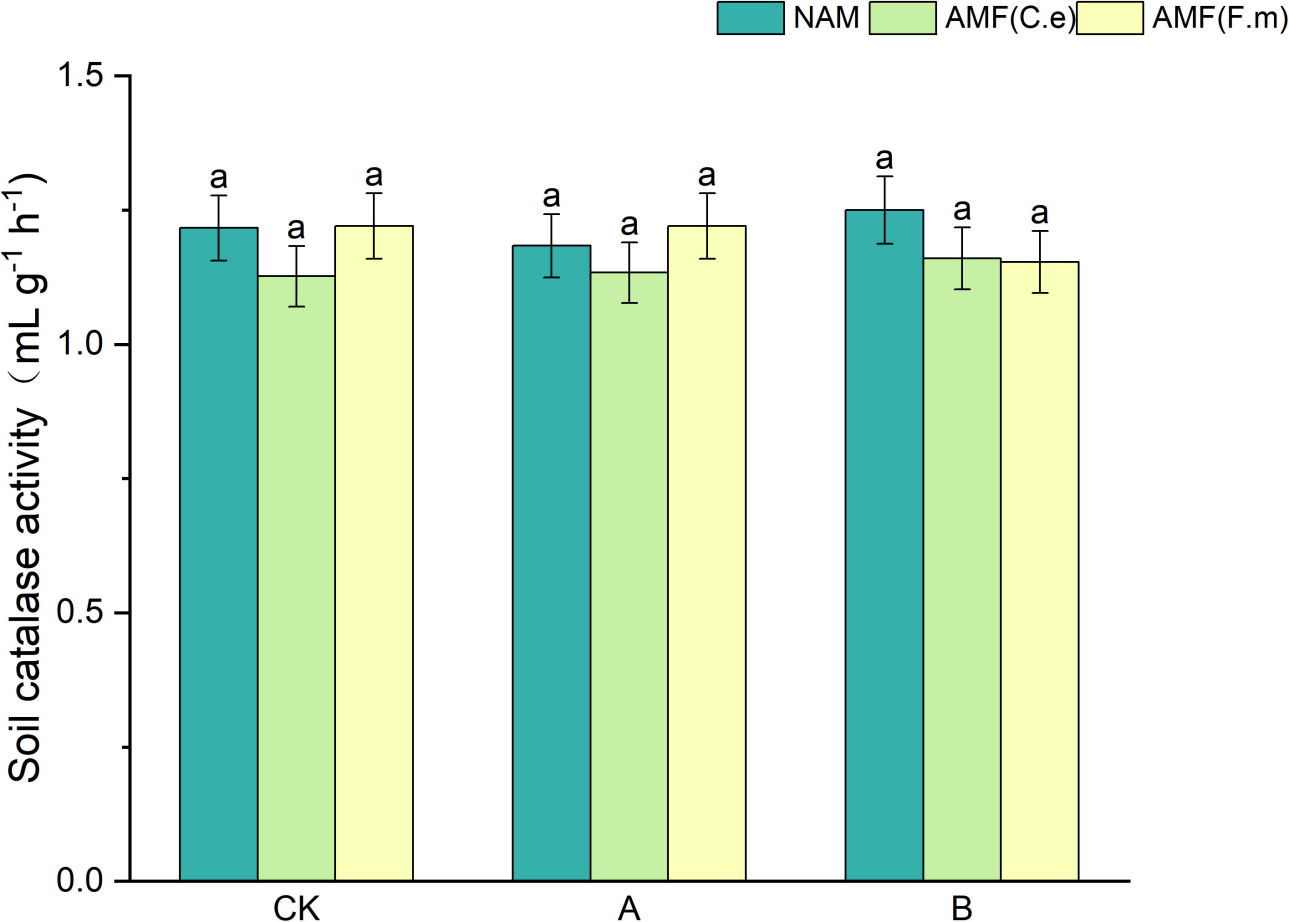
Effects of AMF on soil catalase activity under different nitrogen levels. NAM, no inoculation control; AMF(C.e), inoculation of *Claroideoglomus etunicatum*; AMF(F.m), inoculation of *Funneliformis mosseae*; CK, conventional fertilization; A, reduced fertilization by 30%; B, reduced fertilization by 60%. Data are presented as mean ± standard errors. Different lowercase letters indicate significant differences at the 5% level.

As can be seen from Figure 1, catalase activity decreased and then increased with decreasing fertilizer application, i.e., B > CK > A, at all three levels of fertilization when not inoculated (NAM), as compared to conventional fertilization (CK); Inoculation with AMF (C.e) resulted in unchanged catalase activity at 30% reduction in fertilization (A) and reduced catalase activity at 60% reduction in fertilization (B) as compared to conventional fertilization (CK); Inoculation with AMF (F.m) resulted in unchanged catalase activity at 30% reduction in fertilization (A) and reduced catalase activity at 60% reduction in fertilization (B) as compared to conventional fertilization (CK). The peroxidase activity was reduced when inoculated with AMF (C.e and F.m) compared to non-inoculated (NAM) at conventional fertilization (CK) level; Catalase activity was unchanged when inoculated with AMF (F.m); At 30% reduction fertilization (A), inoculation with AMF (C.e and F.m) resulted in decreased catalase activity when inoculated with AMF (C.e) and elevated catalase activity with 3.28% increase when inoculated with AMF (F.m) as compared to uninoculated (NAM); Hydrogen peroxidase was reduced in both AMF (C.e and F.m) inoculated compared to uninoculated (NAM) at 60% reduction fertilization (B).

### 3.3 Effect of nitrogen fertilizer regulation induced AMF on soil phosphatase activity

**FIGURE 2:**
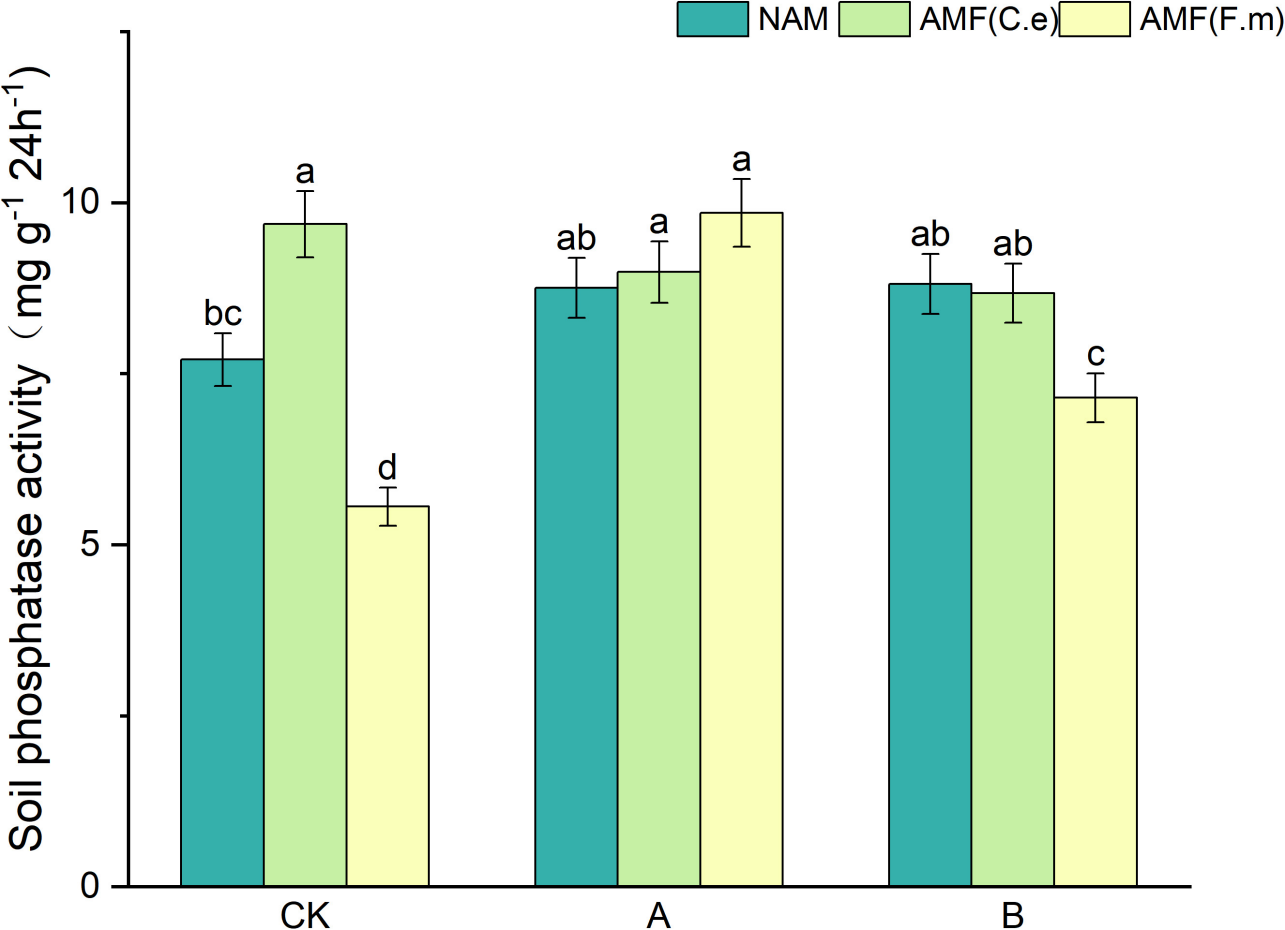
Effects of different fertilization levels of AMF on soil phosphatase activity. See Figure 1 for treatment abbreviations. Data are presented as mean ± standard errors. Different lowercase letters indicate significant differences at the 5% level.

As can be seen in Figure 2, soil phosphatase activity increased with decreasing fertilizer application at three levels of fertilization compared to conventional fertilization (CK) in the absence of inoculation (NAM); When inoculated with AMF (C.e), soil phosphatase activity decreased with reduced fertilizer application compared to conventional fertilization (CK); When inoculated with AMF (F.m), soil phosphatase activity increased with decreasing fertilizer application compared to conventional fertilization (CK). Soil phosphatase activity was elevated and increased by 25.68% when inoculated with AMF (C.e and F.m) as compared to uninoculated (NAM) at conventional fertilization (CK) level and decreased when inoculated with AMF (F.m); At 30% reduction in fertilization (A), catalase activity was elevated in both AMF (C.e and F.m) inoculated with AMF (C.e and F.e) as compared to uninoculated (NAM), which increased by 2.66%, 12.44%, respectively; Soil phosphatases were reduced in AMF (C.e and F.m) inoculated compared to uninoculated (NAM) at 60% reduction fertilization (B).

### 3.4 Effect of nitrogen fertilizer regulation induced AMF on soil urease activity

**FIGURE 3:**
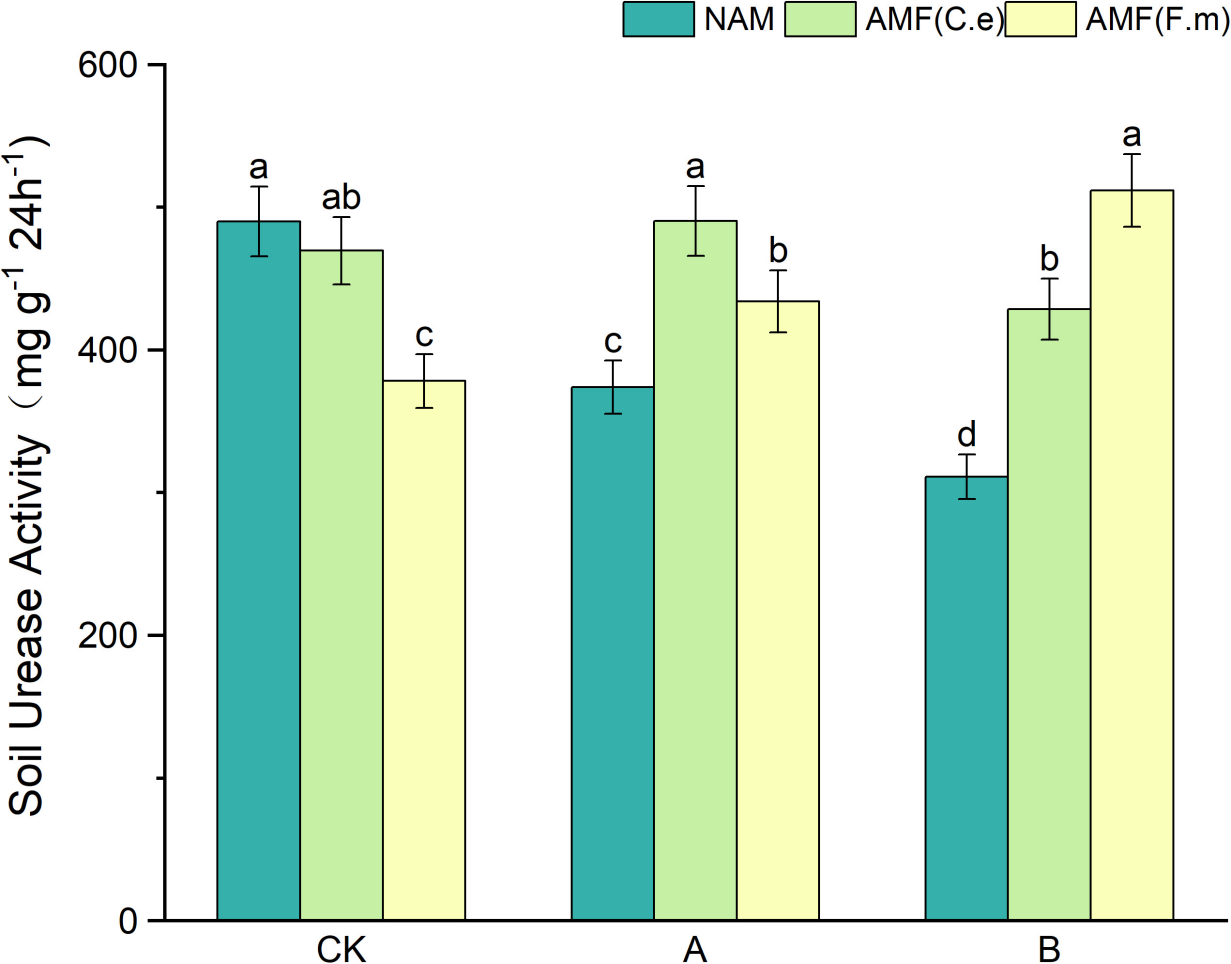
Effects of different fertilization levels of AMF on soil urease activity. See Figure 1 for treatment abbreviations. Data are presented as mean ± standard errors. Different lowercase letters indicate significant differences at the 5% level.

As can be seen in Figure 3, soil urease activity decreased with decreasing fertilizer application at three levels of fertilization compared to conventional fertilization (CK) in the absence of inoculation (NAM); When inoculated with AMF (C.e), soil urease activity increased and then decreased with decreasing fertilizer application, i.e., A > CK > B, as compared to conventional fertilization (CK); When inoculated with AMF (F.m), soil urease activity increased with decreasing fertilizer application compared to conventional fertilization (CK). Soil urease activity was reduced in both AMF (C.e and F.m) inoculated with AMF (C.e and F.m) as compared to uninoculated (NAM) at conventional fertilization (CK) level; Soil urease activity was elevated when inoculated with AMF (C.e and F.m) as compared to uninoculated (NAM) at 30% reduction in fertilizer (A), which increased by 31.11%, 16.03%, respectively; Soil urease activity was elevated in AMF (C.e and F.m) inoculated with AMF (C.e and F.m) as compared to uninoculated (NAM) at 60% reduction in fertilizer (B), which increased by 37.71%, 64.46%, respectively.

### 3.5 Effect of nitrogen fertilizer regulation induced AMF on soil cellulase activity

**FIGURE 4:**
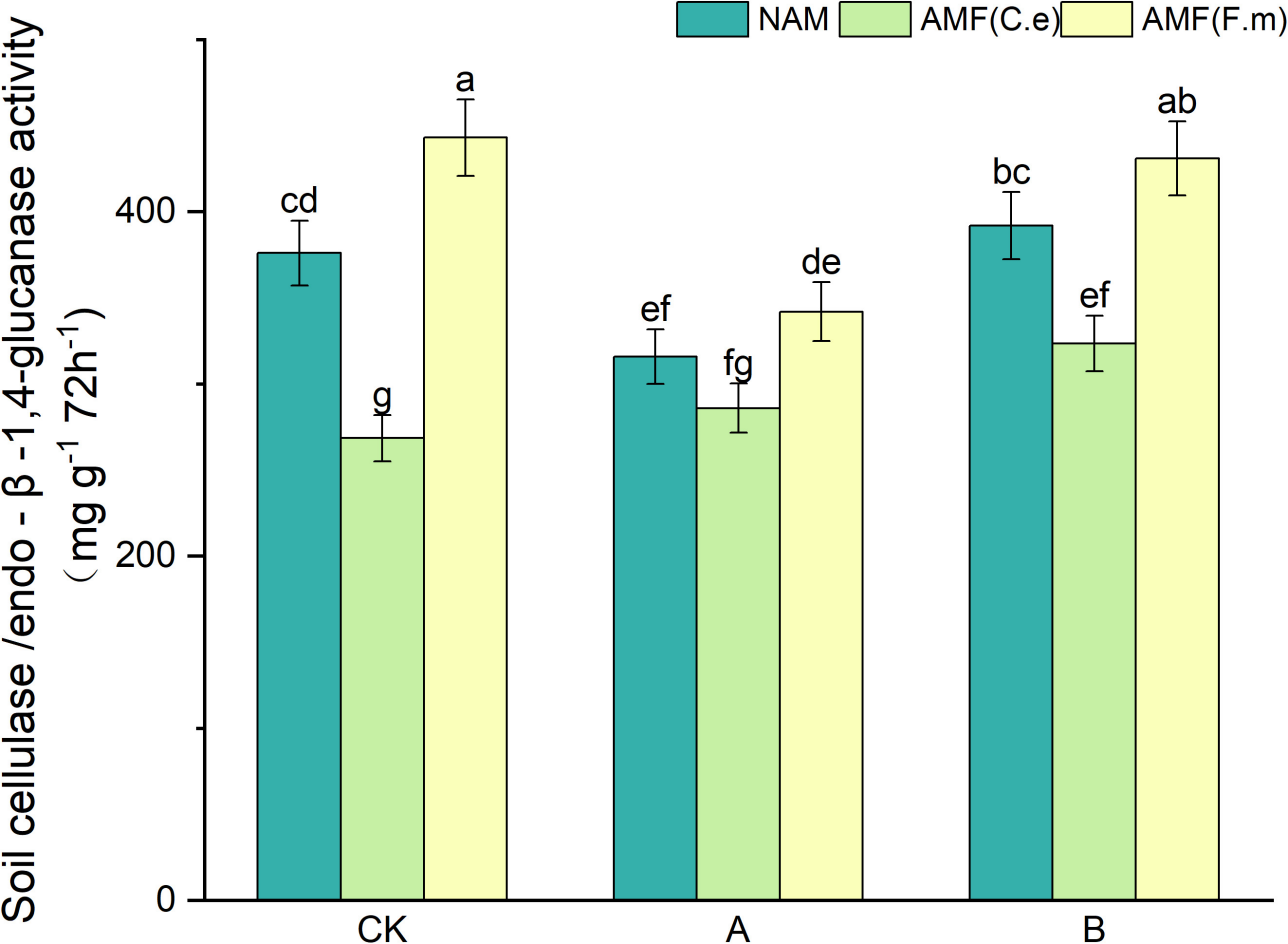
Effects of different fertilization levels of AMF on soil cellulase activity. See Figure 1 for treatment abbreviations. Data are presented as mean ± standard errors. Different lowercase letters indicate significant differences at the 5% level.

As can be seen in Figure 4, soil urease activity decreased and then increased with decreasing fertilizer application, i.e., B > CK > A, at three levels of fertilization without inoculation (NAM) as compared to conventional fertilization (CK); When inoculated with AMF (C.e), soil urease activity increased with decreasing fertilizer application compared to conventional fertilization (CK); when inoculated with AMF (F.m), soil urease activity decreased with decreasing fertilizer application compared to conventional fertilization (CK). Soil urease activity decreased with AMF (C.e) inoculation and increased by 17.8% with AMF (F.m) inoculation as compared to no inoculation (NAM) at conventional fertilization (CK) level; At 30% reduction fertilization (A), inoculation of AMF (C.e and F.m) decreased soil urease activity with AMF (C.e) and elevated soil urease activity with AMF (F.m) by 8.72% as compared to no inoculation (NAM); At reduced 60% fertilization (B), soil urease activity was reduced with AMF (C.e and F.m) inoculation and increased by 9.96% with AMF (F.m) as compared to no inoculation (NAM).

### 3.6 Effect of nitrogen fertilizer regulation induced AMF on soil sucrase activity

**FIGURE 5:**
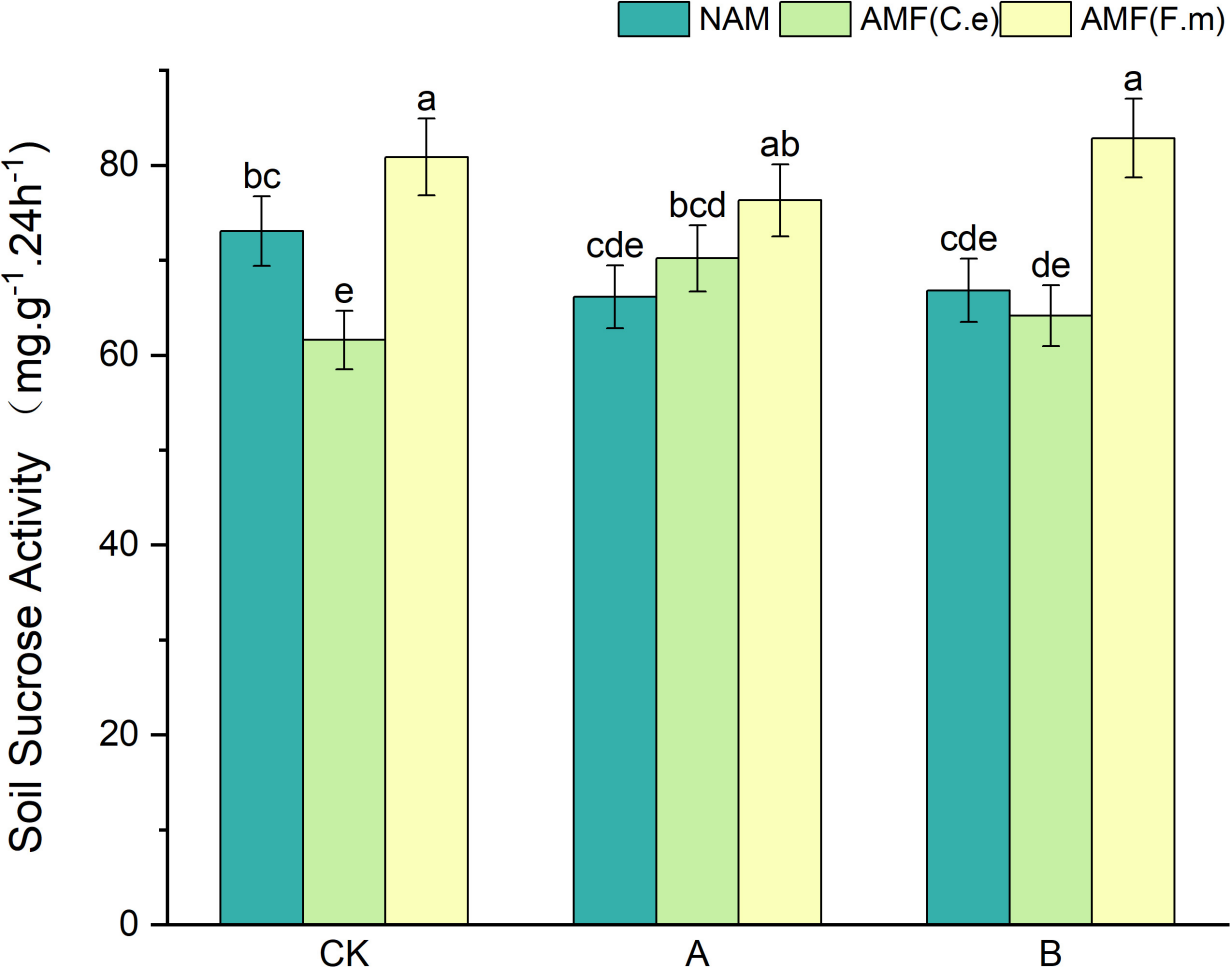
Effects of different fertilization levels of AMF on soil sucrose activity. See Figure 1 for treatment abbreviations. Data are presented as mean ± standard errors. Different lowercase letters indicate significant differences at the 5% level.

As can be seen in Figure 5, soil sucrase activity was reduced at all three fertilization levels without inoculation (NAM), showing a trend of decreasing followed by increasing, i.e., CK > B > A, compared with conventional fertilization (CK); When inoculated with AMF (C.e), soil sucrase activity was increased in all cases, showing a trend of increasing and then decreasing points, i.e., A > B > CK, as compared to conventional fertilization (CK); When inoculated with AMF (F.m), soil sucrase activity decreased and then increased with the decrease in fertilizer application, i.e., B > CK > A, as compared to conventional fertilization (CK). Soil sucrase activity was decreased when inoculated with AMF (C.e and F.m) and increased when inoculated with AMF (F.m) by 10.66%% compared to no inoculation (NAM) at conventional fertilizer (CK) level; at reduced 30% fertilization (A), inoculated with AMF (C.e and F.m) compared to no inoculation (NAM), both soil sucrase activities were elevated when inoculated with AMF (C.e and F.m), which increased by 15.38 and 6.14%, respectively; at 60% reduction fertilizer application (B), soil sucrase activity was reduced when inoculated with AMF (C.e and F.m) compared to uninoculated (NAM) and soil urease activity was elevated when inoculated with AMF (F.m), which increased by 24.02%.

### 3.7 Effect of nitrogen fertilizer regulation induced AMF on plant inter-root soil proteins

**FIGURE 6:**
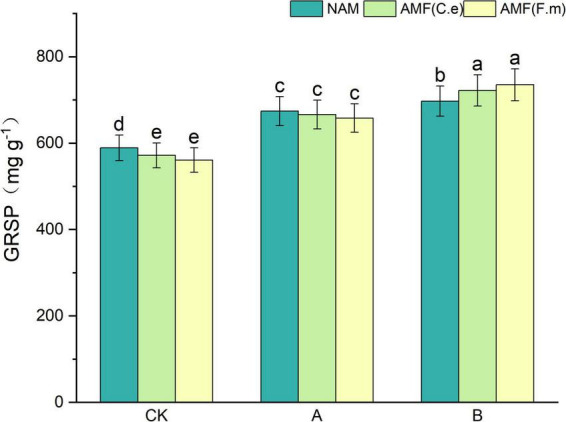
Effect of different fertilization levels of AMF on soil GRSP content. See Figure 1 for treatment abbreviations. Data are presented as mean ± standard errors. Different lowercase letters indicate significant differences at the 5% level.

As can be seen in Figure 6, soil protein (GRSP) content increased with decreasing fertilization compared to conventional fertilization (CK) at three fertilization levels when not inoculated (NAM); when inoculated with AMF (C.e), soil protein (GRSP) content increased with decreasing fertilization compared to conventional fertilization (CK); when inoculated with AMF (F.m), compared to conventional fertilization (CK), soil protein (GRSP) content increased with decreasing fertilizer application. At conventional fertilization (CK) level, the soil protein (GRSP) content was reduced in both AMF (C.e and F.m) inoculated compared to uninoculated (NAM); at reduced 30% fertilization (A), the soil protein (GRSP) content was reduced in both AMF (C.e and F.m) inoculated compared to uninoculated (NAM); at reduced 60% fertilization (B), the soil protein (GRSP) content was reduced in both AMF (C.e and F.m) and uninoculated (NAM); at reduced 60% fertilization (B), the soil protein (GRSP) content was reduced in both AMF (C.e and F.m) and uninoculated (NAM). GRSP) content was decreased in all cases; at reduced 60% fertilization (B), soil protein (GRSP) content was increased when inoculated with AMF (C.e and F.m) compared to uninoculated (NAM), which increased by 3.5%, 5.16%, respectively.

### 3.8 Effect of nitrogen fertilizer regulation induced AMF on the species composition of plant inter-root soil bacteria

**FIGURE 7:**
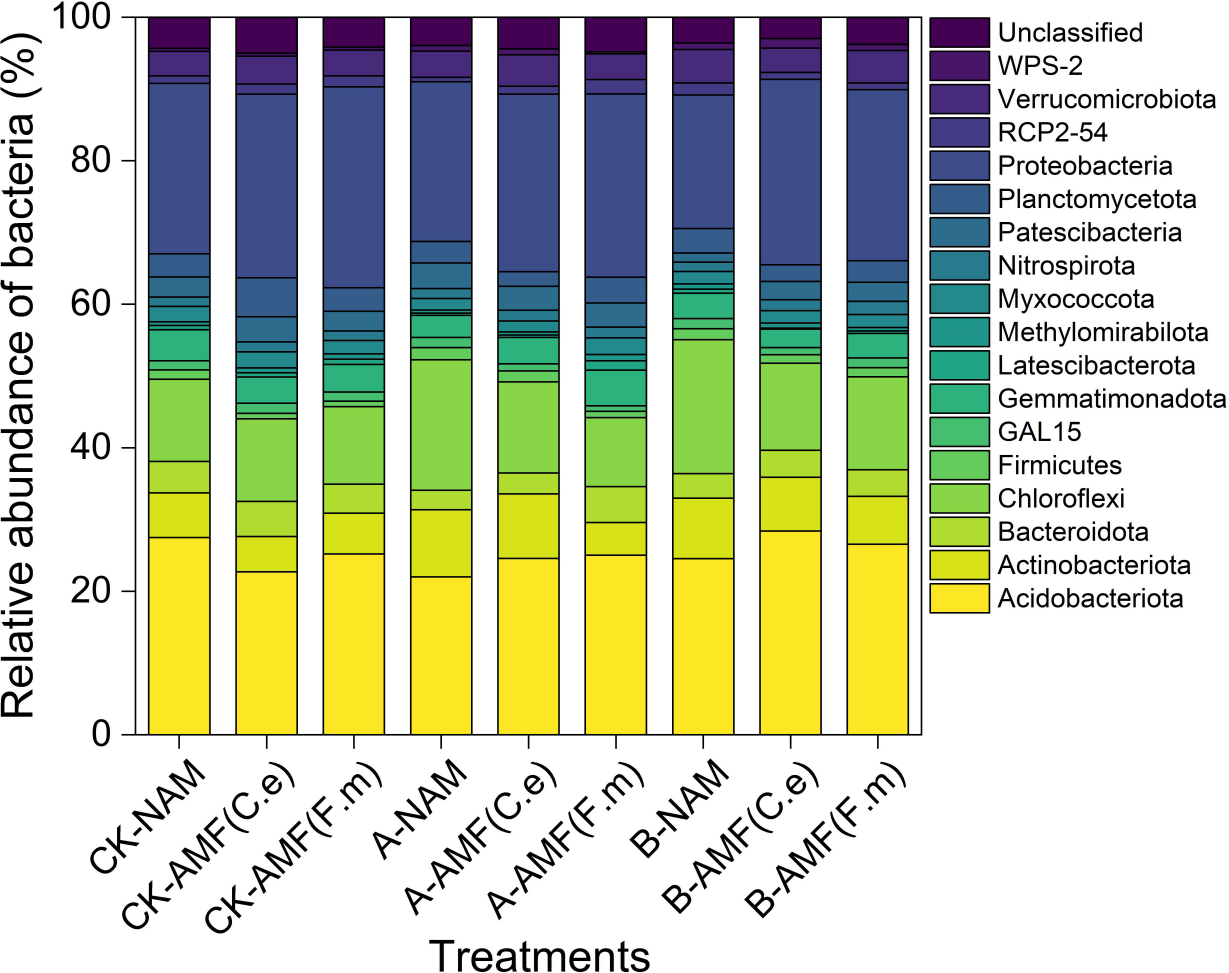
Effects of AMF on the relative abundance of soil bacteria phylum in different fertilization levels.

As illustrated in Figure 7, the analysis was conducted based on high-throughput sequencing data of 27 soil samples, with a primary focus on bacterial phyla exhibiting a relative abundance > 1% (other low-abundance phyla were categorized as “Others”). A total of 18 bacterial phyla were detected, among which four phyla dominated the rhizosphere soil community: Acidobacteriota (25.17%), Proteobacteria (24.24%), Chloroflexi (13.10%), and Actinobacteriota (6.93%). These four phyla accounted for more than 70% of the total bacterial community, indicating their crucial roles in sustaining soil ecological functions. Other phyla with relatively high abundances included Patescibacteria (2.87%), Verrucomicrobiota (3.90%), Gemmatimondota (3.67%), Bacteroidota (3.87%) and Planctomycetota (3.26%).

Compared with conventional fertilization (CK), nitrogen reduction treatments (A and B) led to a decrease in the relative abundances of Proteobacteria and Acidobacteriota. For instance, under 30% nitrogen reduction (A), the relative abundances of Proteobacteria and Acidobacteriota decreased by 6.79 and 25.39%, respectively; under 60% nitrogen reduction (B), the respective decreases were 9.95 and 17.54%. In contrast, the relative abundance of Chloroflexi increased under nitrogen reduction treatments: it rose by 37.52% under 30% nitrogen reduction (A) and by 37.34% under 60% nitrogen reduction (B) compared with CK. Inoculation with arbuscular mycorrhizal fungi (AMF, i.e., C.e and F.m) did not significantly alter the overall composition of the dominant bacterial phyla at the phylum level. Under the same nitrogen level, no consistent trend in the relative abundances of the four dominant phyla was observed between AMF-inoculated treatments and the non-inoculated control (NAM). This figure demonstrates that nitrogen fertilization level is a key driver of changes in the abundance of rhizosphere bacterial phyla. Under nitrogen-limited conditions, the abundances of Proteobacteria and Acidobacteriota decreased significantly, while that of Chloroflexi increased. In contrast, AMF inoculation had a negligible impact on the overall composition of the dominant bacterial phyla.

### 3.9 Effect of nitrogen fertilizer regulation induced AMF on the structure of soil bacterial community between plant roots

**FIGURE 8:**
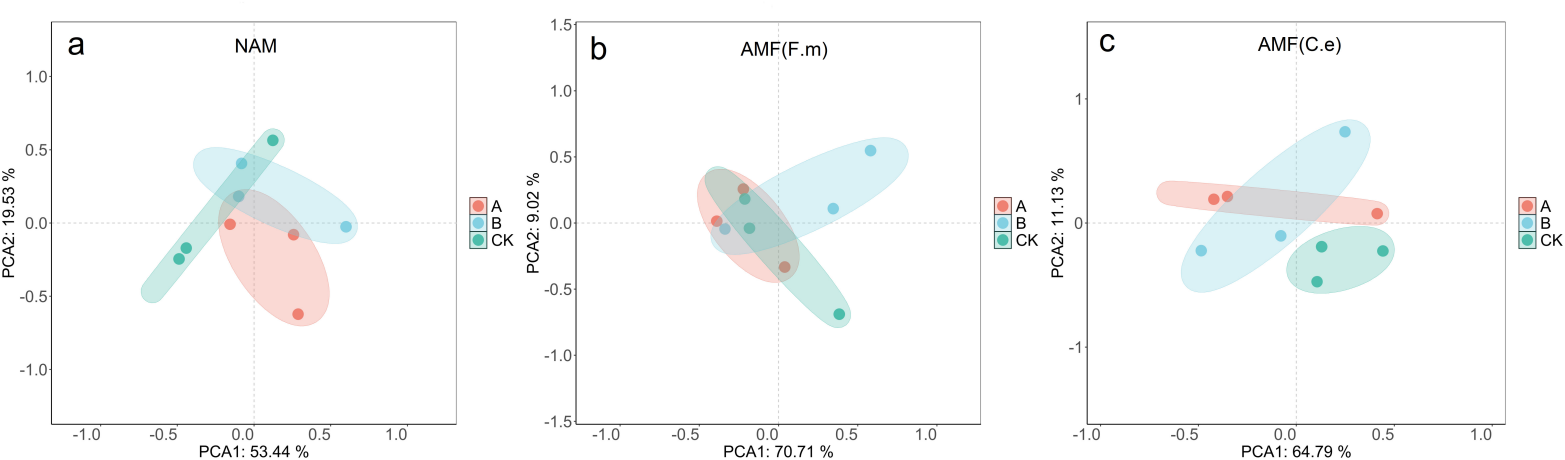
Effect of AMF on bacterial community under different fertilization levels.

As shown in Figures 8a–c, the samples within the same treatment were basically clustered within the confidence ellipses, indicating good repeatability of the microbial community structure. The first principal component (PC1) explained 53.44% – 84.79% of the total variance, which was much higher than that of PC2 (9.02% – 21.95%), suggesting that PC1 was the dominant driving factor for community differentiation. Notably, the samples of different treatments (A, B, CK) showed an obvious separation trend along the principal component axes. For example, in Fig. b, the CK samples were clearly separated from the A and B samples along the PC1 axis, meaning that the applied treatments (such as nitrogen reduction measures) significantly shaped the microbial community structure. The PCA results indicated that the nitrogen level was a key determining factor for the assembly of the rhizosphere bacterial community.

## 4 Discussion and conclusion

### 4.1 Effect of AMF on soil nutrient availability

Soil physicochemical properties are the core foundation of crop growth and soil health. Reasonable fertilization can optimize its structure and fertility, while over-fertilization can easily lead to salinization, acidification and ecological imbalance. AMF plays a crucial role in soil physicochemical properties by improving soil structure and nutrient cycling, enhancing the mineralization process of soil organic matter and the conversion efficiency of nitrogen, thus significantly increasing the effectiveness of nutrients in the soil (Fall et al., 2022). However, this effect varied with fertilization level showing a bidirectional moderating effect with low fertilization promoting nutrient activation, medium fertilization synergistically increasing efficiency, and high fertilization possibly reducing nutrient effectiveness due to competitive inhibition. Therefore, AMF plays an important role in optimizing soil nutrient cycling, and is able to maintain the supply of nutrients to the soil despite reduced nitrogen fertilizer application, providing the necessary nutrient support for plant growth (Ahmed et al., 2025).

The effect of AMF on the physicochemical properties of inter-root soil under different fertilization levels were investigated in this study (Table 1), and the results of the study showed that AMF (C.e) significantly enhanced soil hydrolyzable nitrogen by 26.29% under the 30% nitrogen reduction treatment indicated that AMF (C.e) may directly drive plant nitrogen uptake by optimizing the microenvironment for nitrogen supply in the rhizosphere. Previous studies have demonstrated that AMF (C.e) induce the expression of ammonium transporter (AMT) genes in plant roots. This transporter, localized to the root plasma membrane, can directly mediate the high-affinity transport of ammonium nitrogen (Hui et al., 2022), therefore, inorganic nitrogen is assimilated into glutamine in extraradical hyphae, and subsequently, ammonium is released into the rhizosphere through the arginine metabolic pathway (Hui et al., 2022). Therefore, in the present study, AMF (C.e) significantly increased soil hydrolyzable nitrogen (Table 1), which may include decomposition products of organic nitrogen. Extraradical hyphae of AMF can accelerate the mineralization of organic nitrogen by secreting enzymes such as proteases, thereby releasing ammonium nitrogen for plant uptake (Monika et al., 2022). Furthermore, AMF (C.e) may also promote the dissolution of insoluble organic nitrogen by altering soil pH or secreting organic acids, thereby further enhancing the nitrogen uptake efficiency of plants.

Arbuscular mycorrhizal fungi (F.m) increased organic matter by 10.93% and fast-acting potassium by 2.38%, while had little effect on soil nitrogen in this study may be related to their respective specific metabolic pathways and effects on soil nutrient transformation processes. In the present study, the regulatory pathway of AMF (F.m) in nitrogen metabolism was relatively weak, and its nutrient acquisition strategy may tend to focus on carbon metabolism and potassium uptake rather than directly promoting nitrogen uptake. Under the condition of 30% nitrogen reduction, F.m may not have activated genes related to nitrogen uptake, resulting in a limited direct promoting effect on nitrogen uptake. However, previous studies have shown that AMF (F.m) promote the formation of soil aggregates through the secretion of polysaccharides by their hyphae, thereby increasing the stability and accumulation of soil organic matter (Costa et al., 2018). Additionally, the hyphal network expands the range of carbon sources in the rhizosphere, facilitating the decomposition of organic matter by microorganisms and the subsequent release of organic matter into the soil. Furthermore, the hyphae of AMF (F.m) secrete organic acids to dissolve potassium in soil minerals, and directly promote potassium ion uptake by inducing the expression of plant potassium transporters. In conclusion, the species-specificity of AMF is reflected in their acquisition strategies for different nutrient elements: C.e is more adept at the efficient uptake of nitrogen and phosphorus, whereas F.m focuses on carbon pool management and potassium uptake. This difference stems from variations in their hyphal functions, gene expression patterns, and regulatory pathways for the rhizospheric microenvironment, ultimately leading to functional differentiation under different nitrogen fertilizer reduction conditions.

### 4.2 Regulation of soil enzyme activities by AMF

As key bioactive substances driving biochemical processes in soil ecosystems, soil enzymes directly regulate the efficiency of nutrient conversion and cycling of carbon, nitrogen, and phosphorus (Feng et al., 2025). The results of this study further revealed the role of AMF in regulating the ecological function of soil enzyme system. Under conventional fertilization conditions, AMF (C.e) increases phosphatase activity by 25.68% through two pathways: direct action and microbe-mediated indirect action. At the direct level, organic acids (such as oxalic acid and citric acid) secreted by its mycelium network can chelate metal ions in the soil, alleviating the inhibitory effect of these ions on phosphatase (Fall et al., 2022; Ma et al., 2022). At the same time, spherical sphingomyelin-related soil proteins (GRSPs) produced by AMF (C.e) not only stabilize soil aggregates, but also provide a protective microenvironment for phosphatases, reducing the probability of their degradation by proteases (Rillig and Mummey, 2006). Indirectly, AMF (C.e) enriches specific phosphorus-solubilizing bacteria through mycelial secretions (such as vitamins and amino acids), which in turn secrete additional phosphatases to accelerate organic phosphorus mineralization (Fall et al., 2022). In contrast, in the stress environment with 60% nitrogen reduction, AMF (F.m) significantly increased urease activity by 64.46% by inducing structural reorganization of the inter-root microbial community, and this strain-specific response was closely related to the ecological strategy of the F.m strain to intensify the secretion of carbon sources from the inter-root and to promote the proliferation of urease-producing bacteria under low-nitrogen conditions (Wang et al., 2023).

Further analysis showed that the regulation of enzyme activity by AMF has a dual pathway of influencing the expression of enzyme synthesis genes through mycelial secretion in a direct way and optimizing the structure of enzyme functional community through reshaping the microbial co-occurrence network in an indirect way. Especially under extreme nitrogen reduction conditions, the AMF (F.m) strain showed a stronger environmental adaptability by secreting signaling molecules, it attracts the colonization of urease-producing strains such as *Bacillus*, enhancing urease activity and compensating for nitrogen source supply. Whereas, Under conventional fertilization, AMF (C.e) enriches phosphatase-producing strains such as *Acidithiobacillus*, which decompose organic phosphorus by secreting acidic phosphatase, releasing orthophosphate for plant absorption. This process synergizes with the C.e-induced plant phosphorus starvation response pathway, significantly enhancing phosphatase activity. In summary, different AMF species are specifically adapted to different nitrogen fertilizer conditions. Utilizing the specific adaptability of different strains to various soil enzymes under different soil nutrient conditions will provides an important theoretical basis for the development of mycorrhizal biofertilizers and the construction of low-carbon agricultural system.

### 4.3 Contribution of AMF to soil protein accumulation

Glomalin-related soil protein, as a metal ion-rich hydrophobic glycoprotein secreted by AMF mycelium, is a core component of the soil protein accumulation network, and its secretion directly reflects the contribution of AMF to the soil organic matter pool (Ling et al., 2025). In this study, the nitrogen fertilizer gradient regulation combined with AMF synergistic effect experiment revealed that nitrogen fertilizer reduction combined with AMF inoculation significantly promoted the accumulation of globularin-associated soil proteins (GRSPs), and GRSPs increased by 5.16% under AMF (F.m) treatment. This result is consistent with the findings of previous studies. The increase in GRSP significantly improved soil structure, which promotes the formation of macroaggregates (Rillig, 2004), enhances soil porosity and permeability, improves water and fertilizer retention (Son et al., 2024), and protects organic carbon from rapid decomposition (Son et al., 2021; Zhang et al., 2022). On one hand, the hydrophobic groups of GRSP (e.g., lipids, aromatic amino acids) form a protective hydrophobic layer on the surface of soil particles. This layer reduces the damage to soil aggregates caused by water erosion through hydrogen bonding and van der Waals forces, thereby enhancing the water stability of aggregates. On the other hand, its glycosylated structure binds to soil polysaccharides (e.g., humic acids, extracellular polysaccharides secreted by microorganisms) via ionic bonds and hydrogen bonds, forming stable organic-inorganic composite cementing agents. Meanwhile, F.m induces the proliferation of polysaccharide-producing strains (e.g., Bacillus spp.), which further secrete substances like β-glucan to construct a multi-layered cementing network. The synergistic effect between such hydrophobicity and polysaccharide binding optimizes the particle size distribution of soil aggregates, thereby providing structural support for the ecological intensive cultivation of crops under low-nitrogen environments.

This study also found that GRSP accumulation was closely related to AMF strains and nitrogen reduction levels, suggesting that AMF contributes more significantly to soil carbon sequestration under extreme nitrogen reduction conditions. AMF (F.m) significantly promoted the accumulation of GRSP under reduced nitrogen conditions, probably because it enhances soil structural stability by increasing the secretion of GRSP under restricted nitrogen supply, thus improving the soil’s ability to adsorb and retain nutrients, reducing the risk of nutrient leaching, and safeguarding the sustainability of soil fertility (Deng et al., 2023). It is generally accepted that low-nitrogen conditions induce the proliferation of polysaccharide-producing strains (e.g., Bacillus spp.) associated with AMF (F.m). The polysaccharides secreted by these strains bind to GRSP to form stable cementing agents. Under low-nitrogen environments, F.m regulates the activity of nitrogen metabolism enzymes and prioritizes resource allocation to GRSP production for stress adaptation. In contrast, AMF (C.e) exhibits high efficiency under conventional nitrogen conditions, but its effectiveness diminishes under low-nitrogen conditions. Furthermore, during the late stage of ecological succession, F.m enhances GRSP accumulation through competitive interactions, and its genome may encode a greater number of proteins associated with GRSP secretion. In conclusion, through its secretions, microbial interactions, and metabolic adjustments, F.m demonstrates significantly superior GRSP-secreting capacity compared to C.e under low-nitrogen conditions, thereby more effectively improving soil structure and promoting carbon sequestration.

### 4.4 Effect of AMF on soil microbial communities

The soil microbial community is the most active part of the soil ecosystem, and changes in its composition and structure can have an important impact on soil ecological functions (Hartmann and Six, 2023). The community resolution based on high-throughput sequencing in this study showed that nitrogen fertilization reduction significantly reshaped the composition of the look-alike bacterial community (Figure 7), possibly reflecting the adjustment of microbial ecological niches under different conditions of nutrient effectiveness, e.g., the relative abundance of Proteobacteria (Proteobacteria, oligotrophic r-strategizers) declined by 12.4%, reflecting the abundance of Proteobacteria decreases due to its dependence on high nitrogen environments for nitrogen fixation, and its nitrogenase activity is inhibited, leading to a reduction in denitrification under low nitrogen conditions, and the suppression of its rapid growth metabolism by reduction of effective nitrogen (Yu et al., 2024). The relative abundance of the Chloroflexi, a stress-tolerant chemoenergetic heterotroph, was significantly enriched by 19.7%, revealing Chloroflexi becomes the dominant phylum through efficient carbon fixation via the 3-hydroxypropionic acid pathway under low nitrogen conditions, autotrophic growth using near-infrared light, and degradation of refractory organic matter. Chloroflexi not only promotes the ammonia oxidation activity of other microorganisms by secreting organic acids, thereby enhancing nitrogen conversion efficiency, but its carbon fixation products can also combine with GRSP to enhance soil aggregate stability. Its ecological strategy of adapting to low nutrient stress through mixed nutrient metabolism.

Notably, PCoA analysis in this study showed that the two AMF inoculation treatments did not significantly alter the overall structure of the bacterial community at the gate level, suggesting that AMF may affect soil processes (e.g., nitrogen and phosphorus transformations) primarily by modulating the expression of functional genes (e.g., genes involved in nutrient cycling) in the existing microbial community rather than by drastically altering the composition of the community (Supplementary Table 1; Yu et al., 2025). The above results suggest that in the betel nut intercropped vanilla orchid system, the level of nitrogen fertilizer is the dominant factor in shaping the structure of the inter-root bacterial community in the short term, whereas the role of AMF may be more focused on the fine-tuning of the functional activity or the enrichment of specific functional groups (e.g., nitrogen-fixing bacteria, phosphorus-solubilizing bacteria). This functionally oriented microbial assembly strategy is particularly important under N fertilizer reduction conditions, where AMF achieves precise soil nutrient regulation by optimizing the functional structure of the microbial community rather than simply altering the diversity, highlighting the central role of AMF in maintaining the functional stability of the soil microbiome.

## Data Availability

The datasets presented in this study can be found in online repositories. The names of the repository/repositories and accession number(s) can be found below: doi: 10.17632/fcpwgkts8b.1.
