# Supplementary material for: AMF promotes the structure and metabolic activity of rhizosphere soil microbial communities in areca/vanilla intercropping system under nitrogen-limited conditions
Source: Front Microbiol. 2025 Sep 23;16:1657672. doi: 10.3389/fmicb.2025.1657672 (PMC12500614; doi:10.3389/fmicb.2025.1657672)
Supplement: Supplementary file 1 [file Table_1.docx]

Supplementary Table S1: Relative Abundances of Soil Bacterial Phyla Involved in Nitrogen and Phosphorus Cycling Under Different Treatments

|  | Clusters | | |
| --- | --- | --- | --- |
| Phylum | NAM | AMF(C.e) | AMF(F.m) |
| *Acidobacteriota* | 24.68±2.75^a^ | 25.22±2.89^a^ | 25.60±0.84^a^ |
| *Actinobacteriota* | 8.00±1.63^a^ | 7.15±2.07^a^ | 5.63±1.05^a^ |
| *Bacteroidota* | 3.51±0.84^a^ | 3.86±0.99^a^ | 4.25±0.66^a^ |
| *Chloroflexi* | 16.09±4.02^a^ | 12.10±0.58^a^ | 11.12±1.69^a^ |
| *Firmicutes* | 1.52±0.21^a^ | 1.16±0.40^a^ | 0.98±0.27^a^ |
| *Gemmatimonadota* | 3.65±0.64^a^ | 3.29±0.62^a^ | 4.08±0.83^a^ |
| *Methylomirabilota* | 0.55±0.16^a^ | 0.62±0.15^a^ | 0.69±0.19^a^ |
| *Myxococcota* | 1.83±0.29^a^ | 1.83±0.36^a^ | 2.00±0.27^a^ |
| *Nitrospirota* | 1.33±0.04^a^ | 1.46±0.07^a^ | 1.55±0.26^a^ |
| *Planctomycetota* | 3.21±0.21^a^ | 3.27±1.87^a^ | 3.29±0.28^a^ |
| *Proteobacteria* | 21.53±2.65^b^ | 25.40±0.61^ab^ | 25.80±2.11^a^ |
